# Emotional Valence, Arousal, and Threat Ratings of 160 Chinese Words among Adolescents

**DOI:** 10.1371/journal.pone.0132294

**Published:** 2015-07-30

**Authors:** Samuel M. Y. Ho, Christine W. Y. Mak, Dannii Yeung, Wenjie Duan, Sandy Tang, June C. Yeung, Rita Ching

**Affiliations:** 1 Psychology Laboratories, Department of Applied Social Sciences, City University of Hong Kong, Hong Kong, China; 2 The Women’s Foundation, Hong Kong, China; Zhejiang Key Laborotory for Research in Assesment of Cognitive Impairments, CHINA

## Abstract

This study was conducted to provide ratings of valence/pleasantness, arousal/excitement, and threat/potential harm for 160 Chinese words. The emotional valence classification (positive, negative, or neutral) of all of the words corresponded to that of the equivalent English language words. More than 90% of the participants, junior high school students aged between 12 and 17 years, understood the words. The participants were from both mainland China and Hong Kong, thus the words can be applied to adolescents familiar with either simplified (e.g. in mainland China) or traditional Chinese (e.g. in Hong Kong) with a junior secondary school education or higher. We also established eight words with negative valence, high threat, and high arousal ratings to facilitate future research, especially on attentional and memory biases among individuals prone to anxiety. Thus, the new emotional word list provides a useful source of information for affective research in the Chinese language.

## Introduction

Recent studies have provided unequivocal evidence that emotion and cognitive processes are closely coupled [1]. Various experimental paradigms have been developed to examine how stimuli with emotional content influence cognition. The dot probe task [2] and the emotional Stroop task [3] are most frequently used for investigating attentional processes. The directed-forgetting paradigm [4] and implicit and explicit memory tasks [5, 6] are frequently used to examine memory processes.

Studies such as these may present pictures, faces, or words as emotional stimuli. One advantage of verbal stimuli is the ability to control a series of quantifiable factors (e.g. number of syllables) known to affect word processing, which is not the case for other stimulus materials such as pictures or faces. However, creating highly controlled word-based materials for studying emotional information processing requires not only the careful matching of various factors known to influence word perception, but also a reliable measure of emotional content. For instance, Bradley and Lang [7] produced the Affective Norms for English Words (ANEW), which provide a set of normative emotional ratings for a large number of words. Studies investigating cognitive biases have also provided many appropriate words [8–11]. The availability of such stimuli makes the replication of results and scientific communication easier and comparative studies with different categories of stimuli and sensorial modalities possible. Affective word lists have been developed in other languages apart from English, including Finnish [12], Spanish [13, 14], German [15], Italian [16], European Portuguese [17], and Dutch [18]. Like their English language counterparts, these non-English-language affective word lists have been widely used in memory and perception experiments. For example, Onraedt and Koster [19] used a Dutch affective word database in a study of working memory, whereas Siakaluk, Knol, and Pexman [20] adopted the valence and arousal ratings of affective words as the stimuli in the Stroop task.

Three components of emotion are commonly used [21]: the valence or pleasantness of the stimuli; the arousal or excitement provoked by the stimuli; and the dominance or degree of control exerted by the stimuli. Of these, emotional valence has been identified as the most powerful measure of the emotional nature of stimuli, and has also been shown to capture cognitive resources such as attention [22, 23]. The level of arousal is the second major dimension of emotional affect [7, 24], and there is increasing evidence for its importance [25]. Dominance has been less commonly used [18], and some studies have only included valence and arousal, not dominance [12, 13, 15].

In addition to the above three dimensions, studies on anxiety have found an association between threat-related attentional biases and anxiety [26]. However, although not all negative words are necessarily threatening, the two emotional traits are often mingled. Some studies support the idea that threat captures attention in all individuals only if it exceeds a critical threshold [27, 28]. It would be useful, therefore, to distinguish between threat and negative emotional valence and the level of threat for each word, yet there is a dearth of information in this area. In the current study, ratings on valence and threat (or the potential harm induced by the stimuli) were obtained for each word to provide data on words that are unpleasant (negative), threatening, and high in arousal. Only one study, on spoken French words, has provided ratings of these three dimensions [29]. Nevertheless, threat word cues are important stimuli for studies on cognitive processing and memory biases [9, 30].

Chinese is one of the most widely used languages in the world, yet to the best of our knowledge, no previous study has compared the emotional ratings of Chinese words with ratings of the corresponding words in English norms. Previous studies on Chinese subjects have typically used translated texts and adopted the emotional ratings of English words [31]. However, the polysemy or syntactic ambiguity of a word, or the lack of lexical parallelism between Chinese and English, may undermine the comparability of ANEW’s word ratings. A well-known example is the word “crisis” in English which is depicted by two characters in Chinese *(危機*). The first character *(危)* represents *danger* while the second *(機*) represents *opportunity*. It has been suggested that the word crisis may not have a high negative valence in Chinese [32]. Furthermore, the perception of certain emotional words may change during adolescence. Considering the increased interest in the study of cognitive processing in children and adolescents, and the advantages associated with the use of words in terms of experimental manipulation and control, the aim of the present study was to validate the emotional valence, arousal level, and threat level of Chinese words. Specifically, we aimed to adapt the word stimuli to Chinese at an appropriate reading level for Chinese adolescents. Our findings provide a set of normative emotional ratings for a large number of words in the Chinese language, which will be a useful tool for use in future studies of emotion and cognition conducted among adolescents in Chinese community.

In summary, this study was conducted to (1) establish a list of affective words in Chinese to facilitate cognitive research, especially experiments on working memory and attentional bias for children and adolescents; (2) establish words with threat rating which are less available in the literature; (3) provide ratings of the threat dimension to examine the valence-threat and arousal-threat bi-dimensional relationships; and (4) create a list of words that are highly negative and elicit high arousal and threatening reactions among people for future research.

## Methods

### Participants

The participants were 164 students recruited from two secondary schools in mainland China (n = 102): School A, n = 71, School B, n = 31, and one secondary school in Hong Kong (School C, n = 62). All of the participants were adolescents with no history of psychopathology or psychotic spectrum disorders. All were native Chinese speakers: participants from Hong Kong were able to read traditional Chinese and participants from mainland China were able to read simplified Chinese. As a result, for participants from Hong Kong, traditional Chinese was used for all assessment materials (words for rating, inventories etc.) whereas for participants from Mainland China, simplified Chinese was used. The contents of two Chinese versions of the materials are identical. Our previous studies have confirmed that these two forms of Chinese can be used interchangeably with little effect on the results [33, 34].

### Procedure

Written informed consent from the parents or guardians was first obtained through the school teachers. On the assessment day, students were informed that participation was voluntary and they could withdraw from the study at any time without any negative consequences. Anonymity and confidentiality of participations were assured. Written informed consent was obtained from the students before commencement of the experiment. Ethics approval was obtained from the Human Subjects Ethics Sub-Committee of the City University of Hong Kong.

Rating 300 words on 4 dimensions (valence, arousal, dominance, and threat) would have meant each participant rating 1,200 items, which could have led to fatigue and non-compliance behavior (e.g. missing responses). The following two strategies were adopted to reduce the above issues. First, we excluded dominance as ratings on this dimension are less commonly collected in other studies, according to the literature review. Second, the participants were randomly assigned to one of three groups, each of which rated the words on only one of the three emotional dimensions: 56 (Group 1: mainland China, n = 31; Hong Kong, n = 25) rated the emotional valence of words; 53 (Group 2: mainland China, n = 37; Hong Kong, n = 16) rated the emotional arousal of the same words; and another 51 (Group 3: mainland China, n = 30; Hong Kong, n = 21) rated the threat value of the same words. Gender and age were balanced across groups. As a result, each participant only needed to rate 300 items on one emotional dimension. This arrangement could also minimize the possibility of mutual influence between the emotional valence, arousal, and threat ratings.

The participants in groups 1 and 2 rated the emotional valence and arousal levels of the words, respectively, on a 9-point Self-Assessment Manikin scale (SAM) [35, 36]. The participants in group 3 rated the threat levels of the words on a five-point scale. Details of these rating scales are provided in the Measures section below. A paper-and-pencil procedure was adopted for the affective rating task. The data were collected in the participants’ classrooms. In each session, before the data collection, a research assistant with a psychology educational background presented the aim of the study to the students, and emphasized the voluntary nature of their participation and the confidentiality of the results. Students were allowed to drop out of the study at this stage if they did not want to participate. Subsequently, the research assistant explained the affective rating task by describing the use of the SAM scale to groups 1 and 2, and the use of the threat-value scale to group 3. The participants were reminded that a personal, subjective rating was required, and thus there were no correct or incorrect answers. The participants were also told that they could mark a specific response if they did not know the meaning of any of the presented words. Before the start of the assessment, six words (love, boat, bomb, duck, trust, and crime) were used as examples to give the participants a basic reference, and an additional self-created word was use to show how to respond in case the word was not comprehensible to them. Finally, questionnaire sets with the word list were distributed to each subject. No time limit was defined, but the subjects were encouraged to answer as quickly as possible. The entire process lasted for about 40 minutes.

### Materials

We first selected 300 English verbs and nouns with reference to the ANEW database [36] and the word lists of previous studies investigating cognitive biases, which provide a source for words pertaining to social and psychological threat [7, 9, 30, 37, 38]. The 300 words comprised 104 negative, 73 positive, and 123 neutral words. A bilingual translator (native Chinese language speaker) with a psychology education background performed the forward translation from English to Chinese, and another bilingual individual with a psychology education background independently translated it back into English. To ensure the items in both versions achieved grammatical and colloquial appropriateness, differences between the original and back-translated versions were discussed and resolved with the agreement of both translators and the first author of this paper. Two versions of the same word list were created with the items presented in random order to exclude the influence of primacy and recency effects on the participants’ ratings.

### Measures

#### Valence and Arousal Scales

The valence and arousal rating scales of the Self-Assessment Manikin (SAM) [7, 35] were used to measure valence and arousal. The SAM consists of two 9-point bipolar ratings scales (ranging from “1” to “9”) with pictorial manikins representing the values on each dimension. In the present study, on the valence scale, “1” was accompanied by a frowning, unhappy figure representing extremely unpleasant, and “9” was accompanied by a smiling, happy figure representing extremely pleasant. On the arousal scale, “1” was accompanied by a relaxed, sleepy figure corresponding to feeling very calm, and “9” was accompanied by an excited, wide-eyed figure corresponding to feeling very excited and aroused. The presentation of the scales was based on empirical evidence that higher numbers intuitively go with positive anchors (e.g. sad to happy rather than happy to sad) [39].

#### Threat-value Scale

As in the study by Bertels, Kolinsky, and Morais [29], words were rated according to their threat value on a 5-point scale ranging from “1 = not threatening” to “5 = very threatening.”

#### Personal Data

Participants were also asked to provide demographic information (e.g., sex, age, education grade, country/place resided in most between birth and age 7) and answered questions about their language history (e.g., native language, second languages learned), handedness (right-handed, left-handed) and vision (normal, corrected to normal visual acuity). Participants were also asked to indicate whether they knew the meaning of each word (“yes” or “no”).

## Results

### Participant profile

Four participants (2.4%) with more than 10% missing data were excluded from the data analysis, thus the final sample consisted of 160 participants. The participants’ demographic characteristics are presented by school in Table 1.

**Table 1:** Words with High Threat Rating.

Word No.	English Translation	Chinese Word	Threat (Rating = 1–5)	Valence (Rating = 1–9)	Arousal (Rating = 1–9)
			Mean (SD)	Mean (SD)	Mean (SD)
**3**	**Annoyed**	**煩惱**	**3.06 (1.39)**	**2.55 (2.04)**	**5.54 (2.29)**
**5**	**Assault**	**毆打**	**3.36 (1.45)**	**2.47 (1.87)**	**5.53 (2.74)**
**11**	**Beating**	**痛打**	**3.36 (1.34)**	**2.65 (2.02)**	**5.69 (2.59)**
**20**	**Cancer**	**癌症**	**3.57 (1.51)**	**2.27 (1.67)**	**5.82 (2.49)**
28	Collapse	倒塌	3.02 (1.61)	3.55 (2.16)	4.94 (3.00)
**51**	**Dying**	**垂死**	**4.00 (1.23)**	**2.50 (1.84)**	**5.48 (2.63)**
53	Emergencies	緊急	3.35 (1.65)	3.15 (1.88)	4.69 (2.94)
70	Hazard	危險	3.20 (1.53)	3.82 (1.82)	5.30 (2.70)
**77**	**Horror**	**驚駭**	**3.13 (1.48)**	**2.69 (1.75)**	**5.50 (2.54)**
81	Idiotic	白痴	3.34 (1.61)	2.48 (1.90)	4.85 (2.40)
118	Pounding	猛擊	3.50 (1.30)	3.29 (2.28)	5.14 (2.38)
**138**	**Suffocate**	**窒息**	**3.84 (1.36)**	**2.84 (1.84)**	**5.54 (2.72)**
**140**	**Surgery**	**手術**	**3.90 (1.33)**	**2.54 (2.04)**	**6.00 (2.61)**
144	Teased	取笑	3.08 (1.54)	3.15 (2.30)	4.72 (2.69)
155	Violence	暴力	3.06 (1.39)	3.13 (2.26)	5.32 (2.65)

**Note:** High threat rating is defined as a mean score above 3 on a 5-point scale; High negative valence is defined as a mean score below 3 on a 9-point scale; and high arousal is defined as a mean score above 5 on a 9-point scale. High threat, high negative and high arousal words are highlighted in bold fonts.

On average, the Hong Kong students were older than the mainland Chinese students, t(1) = -3.53, p < .01, and the Hong Kong sample had fewer girls than the sample from mainland China, χ²(1) = 4.24, p < .05.

### Selection of Words

Ten words were excluded from the analysis because 10% or more of the participants (ranged from 10% to 15%) stated that they did not understand their meaning. The remaining 290 words were categorized into positive, neutral, or negative valence according to the rating scores of the 56 participants assigned to the valence rating group (see Procedure section) based on the criteria of Ferre et al. [13]: less than 4 = negative; 4 to 6 = neutral; 6 to 9 = positive. The resulting valence classification (positive, negative, neutral) of each word was then compared to its *a priori* classification according to previous studies (see Materials section). Only words that were rated the same in both classifications were selected for further analysis. For example, the word “crazy” (瘋狂 in Chinese) was classified as negative in English according to previous studies but positive in Chinese according to the ratings of the present sample. This word was excluded from the final list. This strategy ensured that the Chinese words in our final list had identical valence to the existing word lists in other languages to facilitate communication and comparison of results. Table 2 depicts the concordance rate of the three valence types. There were 160 words with valence identical to previous studies: 25 positive (15.6%), 90 neutral (56.3%), and 45 (28.1%) negative. These 160 words were included in our final word list.

**Table 2:** Concordance rate by valence of words.

		Classification based on Rating of the Present Study		
		Positive n (%)	Neutral n (%)	Negative n (%)
Classification based on previous Studies	Positive	**25 (8.6)**	47 (16.2)	0
	Neutral	9 (3.1)	**90 (31.0)**	19 (6.6)
	Negative	1 (0.3)	54 (18.6)	**45 (15.5)**

**Note:** Number of words = 290; number of participants did the rating = 56.

### Descriptive Statistics

After categorizing the words into positive, negative, and neutral valence, we categorized them as high or low arousal and high or low threat, according to the following strategies:

1. words with a mean arousal rating score above 5.0 according to the 9-point SAM scale were categorized as high arousal words; and
2. words with a mean score above 3.0 on the 5-point threat rating scale were categorized as high threat words.

According to the above strategies, 20 words (12.5%) were categorized as high arousal and 15 words (9.4%) as high threat. As threat ratings are not commonly available in the literature, we provide the means and standard deviation of the high threat words in Table 3. An Excel file with the data for the 160 words and their classification is available as a supporting information of the article. The top three highest-rated threat words were dying (mean = 4.00, SD = 1.23), surgery (mean = 3.90, SD = 1.33), and suffocate (mean = 3.84, SD = 1.36).

**Table 3:** Words with High Threat Rating.

Word No.	English Translation	Chinese Word	Threat (Rating = 1–5)	Valence (Rating = 1–9)	Arousal (Rating = 1–9)
			Mean (SD)	Mean (SD)	Mean (SD)
**3**	**Annoyed**	**煩惱**	**3.06 (1.39)**	**2.55 (2.04)**	**5.54 (2.29)**
**5**	**Assault**	**毆打**	**3.36 (1.45)**	**2.47 (1.87)**	**5.53 (2.74)**
**11**	**Beating**	**痛打**	**3.36 (1.34)**	**2.65 (2.02)**	**5.69 (2.59)**
**20**	**Cancer**	**癌症**	**3.57 (1.51)**	**2.27 (1.67)**	**5.82 (2.49)**
28	Collapse	倒塌	3.02 (1.61)	3.55 (2.16)	4.94 (3.00)
**51**	**Dying**	**垂死**	**4.00 (1.23)**	**2.50 (1.84)**	**5.48 (2.63)**
53	Emergencies	緊急	3.35 (1.65)	3.15 (1.88)	4.69 (2.94)
70	Hazard	危險	3.20 (1.53)	3.82 (1.82)	5.30 (2.70)
**77**	**Horror**	**驚駭**	**3.13 (1.48)**	**2.69 (1.75)**	**5.50 (2.54)**
81	Idiotic	白痴	3.34 (1.61)	2.48 (1.90)	4.85 (2.40)
118	Pounding	猛擊	3.50 (1.30)	3.29 (2.28)	5.14 (2.38)
**138**	**Suffocate**	**窒息**	**3.84 (1.36)**	**2.84 (1.84)**	**5.54 (2.72)**
**140**	**Surgery**	**手術**	**3.90 (1.33)**	**2.54 (2.04)**	**6.00 (2.61)**
144	Teased	取笑	3.08 (1.54)	3.15 (2.30)	4.72 (2.69)
155	Violence	暴力	3.06 (1.39)	3.13 (2.26)	5.32 (2.65)

**Note:** High threat rating is defined as a mean score above 3 on a 5-point scale; High negative valence is defined as a mean score below 3 on a 9-point scale; and high arousal is defined as a mean score above 5 on a 9-point scale. High threat, high negative and high arousal words are highlighted in bold fonts.

As discussed previously, not all negative words are necessarily threatening, and vice versa. The participants rated 30 words (18.8%) as negative but not as posing a social or psychological threat; for instance, avoid, unkind, ashamed, stupid, embarrass, and lazy. Conversely, all 15 words that were rated as high threat were also rated as negative words. Four words (2.5%) were classified as high threat but low arousal: collapse, idiotic, emergencies, and teased. Nine words (5.6%) were rated as high arousal but low threat: ashamed, stupid, embarrass, reject, terrific, terrified, despise, worried, and coward. Finally, 11 words (6.9%) were rated as high threat and high arousal: hazard, suffocate, assault, annoyed, beating, dying, pounding, cancer, horror, surgery, and violence (Table 4).

**Table 4:** Threat by Valence and Arousal Groups (total number of words = 160).

	Low Threat	High Threat	Total
	n (%)	n (%)	n (%)
**Valence**			
** Positive**	115 (71.9)	0	115 (71.9)
** Negative**	30 (18.8)	15 (9.4)	45 (28.1)
**Arousal**			
** Low**	136 (85.0)	4 (2.5)	140 (87.5)
** High**	9 (5.6)	11 (6.9)	20 (12.5)
**Total**	145 (90.6)	15 (9.4)	160 (100)

It is useful to develop lists of words that are highly negative, and elicit high arousal and threatening reactions among people. We selected words with average scores below 3 on the valence scale (very negative/unpleasant; 9-point scale), above 5 on the arousal scale (high arousal; 9-point scale), and above 3 on the threat scale (high threat, 5-point scale). Eight words fulfilled the above criteria; representing 17.8% of the negative words on our list (see Table 3).

### Relationships between demographic characteristics and ratings

The means and standard deviations of each emotional dimension by gender and location are depicted in Table 5. The mean ratings for valence and arousal were 4.59 (SD = .62) and 4.21 (SD = 1.20) respectively on a 9-point scale whereas those for threat was 2.20 (SD = .62) on a 5-point scale. Gender (male versus female) and region (Hong Kong versus mainland China) did not affect the ratings, except that the mean valence ratings were higher for participants from Hong Kong than for their mainland counterparts, t(54) = -3.87, p< .01. One-way analysis of variance (ANOVA) revealed no differences in the three emotional dimensions between participants of different ages: positive, F(5,144) = 1.13, *p* = .*35*; neutral, F(5,144) = 1.02, *p* = .*41*; and negative, F(5,144) = 1.88, *p* = .*10*.

**Table 5:** Mean (Standard Deviation) for Valence, Arousal, and Threat Ratings by Gender, and Region (number of words = 160).

		Gender			Region		
Type of Words	All	Female	Male	*t* (*df*)	Hong Kong	Mainland China	***t* (*df*)**
Valence Mean (SD) on a 9-point rating scale							
**All**	**4.59 (0.62)**	**4.59 (0.55)**	**4.58 (0.70)**	**-0.06 (54)**	**4.91 (0.36)**	**4.33 (0.68)**	**-3.87 (54)** **
Negative	3.05 (1.08)	3.02 (1.03)	3.07 (1.14)	0.17 (54)	3.45 (0.91)	2.73 (1.11)	-2.61 (54)*
Neutral	4.89 (0.73)	4.95 (0.65)	4.84 (0.81)	-0.53 (54)	5.19 (0.44)	4.65 (0.83)	-2.91 (54)**
Positive	6.55 (1.15)	6.45 (1.13)	6.64 (1.19)	0.62 (54)	6.81 (0.92)	6.34 (1.29)	-1.53 (54)
Arousal Mean (SD) on a 9-point rating scale							
**All**	**4.21 (1.20)**	**4.21 (1.17)**	**4.21 (1.29)**	**0.02 (51)**	**4.06 (1.65)**	**4.27(0.97)**	**0.57 (51)**
Negative	4.79 (1.43)	4.97 (1.40)	4.46 (1.47)	-1.25 (51)	4.94 (1.72)	4.73 (1.31)	-0.50 (51)
Neutral	3.75 (1.37)	3.67 (1.32)	3.88 (1.50)	0.53 (51)	3.55 (1.72)	3.84 (1.21)	0.71 (51)
Positive	4.29 (1.47)	4.20 (1.54)	4.46 (1.36)	0.63 (51)	3.89 (2.21)	4.47 (0.99)	1.34 (51)
Threat Mean (SD) on a 5-point rating scale							
**All**	**2.20 (0.65)**	**2.27 (0.63)**	**2.14 (0.68)**	**-0.68 (49)**	**2.02 (0.66)**	**2.33 (0.62)**	**1.71 (49)**
Negative	2.79 (0.75)	2.85 (0.72)	2.74 (0.78)	-0.55 (49)	2.75 (0.78)	2.82 (0.74)	0.36 (49)
Neutral	2.02 (0.74)	2.09 (0.67)	1.96 (0.80)	-0.61 (49)	1.77 (0.77)	2.20 (0.67)	2.11 (49)*
Positive	1.58 (0.62)	1.64 (0.59)	1.53 (0.65)	-0.61 (49)	1.37 (0.50)	1.73 (0.65)	

* *p* < .05

**p < .01

### Reliability

We adapted the method of Moors and colleagues [18] in a similar study to calculate the reliabilities for each sample of valence (n = 56), arousal (n = 53), and threat (n = 51) ratings separately (see also [21]). As mentioned before (see Procedure), participants were allocated to one of three groups with participants in each group provided ratings on one of the three emotional dimensions (valence, arousal, or threat). Accordingly, participants in each rating group were split into halves according to their serial numbers (odd or even). Interclass correlation coefficients [40] were high for valence (.98), arousal (.84), and threat (.96). We also split each rating group according to gender and location. All reliabilities were good: gender (male versus female): valence (.98), arousal (.84), threat (.96); location (mainland China versus Hong Kong): valence (.98), arousal (.76), threat (.92).

### Bi-directional Relationships between the Variables

The Pearson’s zero-order correlation coefficients between the emotional words are shown in Table 6. There were significant correlations for all of the bi-dimensional relationships in the expected directions. First, valence and threat were negatively correlated (r = -.79, p. < .001), showing that words that were more pleasant were less threatening. Second, a significant negative correlation was obtained between arousal and threat (r = .62, p < .001), i.e. words that were more threatening tended to arouse more excitement among the participants. Finally, a negative linear relationship was obtained between valence and arousal (r = -.43, p < .001), suggesting that words that were more negative tended to elicit more excitement.

**Table 6:** Correlations between the variables.

	Valence	Arousal	Threat
**Valence**	1	-.43**	-.79**
**Arousal**		1	.62**
**Threat**			1

** p < .001

Bradley and Lang [7] and other studies of affective word adaptation in different languages, such as Spanish [13], European Portuguese [17], and Finnish [12] have reported a quadratic relationship between valence and arousal. This quadratic relationship was thus examined in the current study.A U-shaped distribution (R = .72, p < .000), indicating that very positive or very negative words tended to arouse more excitement among the participants, was obtained. This quadratic relationship also explained more of the variance (52.5%) than the linear relationship (18.6%), showing that it fit the data better than the linear model. The same U-shaped distribution was obtained in the abovementioned studies [7, 12, 13, 17].

## Discussion

We establish valence, arousal, and threat ratings for a list of 160 Chinese affective words in this study. As mentioned before (see Procedure section), rating 300 words on 4 dimensions (valence, arousal, dominance, and threat) might lead to fatigue and non-compliance behavior. We have excluded power/dominance in the present study for the following reasons. First, power/dominance has been less commonly used and excluded in previous studies [12, 13, 15, 18]. Second, it is our interest to explore words that are threatening but not negative and vice versa (see Table 3). Finally, we are motivated to establish a list of words that are highly negative, and elicit high arousal and threatening reactions among people for future research (see also Table 3). The words have good reliability, as demonstrated by the high split-half reliability in the three categories: valence (.98), arousal (.84), and threat (.96). To the best of our knowledge, this is the first empirically established affective word list in Chinese. Our word list has several characteristics.

First, we attempted to ensure that the valence classification (i.e., positive, neutral, or negative) of each of our Chinese words corresponded with the classification of their English counterparts in previous studies [7, 30, 37, 38]. For example, the word confident (自信 in Chinese) was classified as positive both in our study (mean valence rating = 6.37, SD = 2.10) and in the ANEW manual [7](mean valence rating = 7.98, SD = 1.29). Similarly, the word despise (鄙視 in Chinese) was categorized as negative both on our list and the ANEW list (our sample: mean = 3.16, SD = 2.25; ANEW: mean = 2.03, SD = 1.38). Due to the lack of lexical parallelism between the Chinese and English languages, we contend that words translated from English into Chinese may not necessarily have the same valence. Our present findings, in fact, support this proposition. For instance, the word crazy was classified as a negative word in English according to a previous study [9]. However, the Chinese translation of this word (瘋狂) carries the connotation of elation or ecstasy, and the young people in our sample regarded it as a positive word. As expected, some words that were classified as neutral in English were considered as positive or negative in Chinese and vice versa. For example, Stewart and colleagues [30] classified the word motel (旅館 in Chinese) as neutral, but our participants considered it a positive word, perhaps because they associated it with traveling and holidays. Forty-seven words (16.2%, Table 2) with positive valence in English were rated as neutral words by our participants. A notable example is the word happiness (快樂 in Chinese), which was classified as positive in McCabe’s [9] study, but as neutral in the present study. The reason for this difference may be that the word happiness carries a hedonic connotation that may not be regarded as positive in Chinese culture [41]. Finally, 54 words (18.6%) that were classified as negative in English were rated as neutral in the current study. Many of these words have marginal ratings in the ANEW database [7], for example, anxious (ANEW valence mean rating = 4.80) and shy (ANEW valence mean rating = 4.59). Our Chinese participants might also regard shy and anxious as adaptive reactions in some situations because there are notable differences in pro-social behavior between Western and Chinese cultures [41–43]. A detailed discussion of the possible reasons for the differences in classification between our findings and those of previous studies is beyond the scope of this paper. We attempted to synchronize the valence of our Chinese words with existing English language word lists, which is an important step in ensuring the consistency of valence across languages, although this attempt reduced the number of words in our list.

Second, our study is one of the few to provide ratings of the threat of emotional words. Highly threatening stimuli are important tools in experimental studies of cognitive biases, especially studies related to the etiology of anxiety [2, 26, 44, 45] and intervention strategies to modify the attentional bias of anxiety prone adolescents [46–49]. Our list contained 15 words that the adolescents perceived as eliciting social and psychological threat, and eight of these were rated highly on threat, arousal, and negative valence: annoyed (煩惱); assault (毆打); beating (痛打); cancer (癌症); dying (垂死); horror (驚駭); suffocate (窒息); and surgery (手術). All of these except horror are related to physical suffering and pain, which may be related to adolescents’ concerns about their physical well-being and appearance at this stage [50]. These words should be helpful for use in experiments on cognitive biases related to anxiety, particularly attentional and memory biases, among adolescents. Our results show that high arousal words do not necessarily have high threat values. For instance, the word terrific (優秀 in Chinese) was not considered as a high threat word, although it had a high arousal value, and also had a high positive valence rating (mean = 6.19, SD = 2.47). Similarly, not all negative words were rated as threatening. Words such as avoid (逃避), ashamed (羞恥), and embarrassed (尷尬) are obviously negative words, but the adolescents in our sample did not appraise them as possessing a high potential to harm.

Third, the words in our list were comprehensible to the majority (> 90%) of our 12- to 17-year-old secondary school participants. There are two forms of written Chinese: simplified and traditional. The former is used mainly in mainland China and Singapore whereas the latter is used in Hong Kong and Taiwan. These two forms of written Chinese have slightly different lexical and grammatical structures, and some expressions and words in simplified Chinese may not be comprehensible to people using traditional Chinese and vice versa. It is important to have a list of emotional words applicable to adolescents familiar with either of the two written forms of Chinese to facilitate comparison across communities. Furthermore, our words were highly comprehensible to junior secondary students, thus it is logical to expect older people with junior secondary education or above (e.g. senior secondary school students, university students, and adults) to understand the meanings of these words. This means that our emotional word list can be applied to people of different age groups, although the valence, arousal, and threat ratings of the words may need to be verified again in future studies.

**Fig 1:**
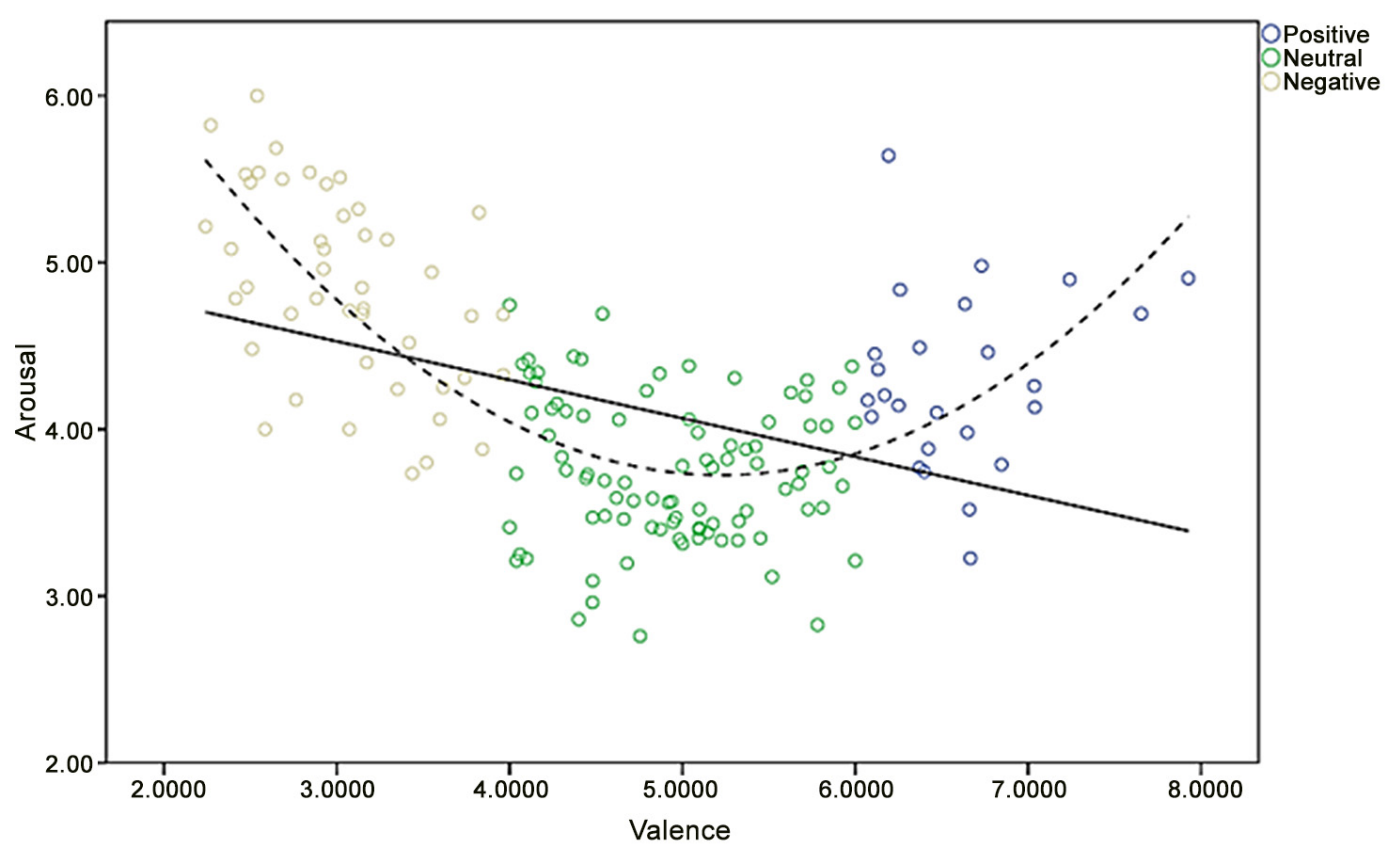
Distribution of the mean values (positive, negative, and neutral types of words) for the ratings of the 160 Chinese words in valence and arousal dimensions.

Moors and colleagues [18] did not find a significant correlation between valence and arousal scores (r = -.01). Although we found a significant linear relationship between these two variables in our study (r = -.43, p < .01), a U-shaped pattern fit our data significantly better than a linear relationship (Fig 1). Consistent with other studies [7, 12, 13, 17, 21], words that are very positive or very negative tended to arouse more excitement among our adolescent participants. As expected, more negative words tended to arouse more threat (r = -.79, p < .01) and more threatening words elicited more arousal (r = .62, p < .01). Finally, the correlational results (Table 6) showed that more negative words tended to be perceived as more threatening while more threatening words tended to elicit more arousal, as would be expected.

In sum, we have developed a list of emotional Chinese words with good reliability and with valence classifications that are consistent with emotional word lists in English. The emotional words were comprehensible to individuals with junior secondary education who read either Simplified or Traditional Chinese characters. We collected threat ratings in addition to valence and arousal ratings, which are available as supporting information (S1 Table) in MS Excel format. However, further studies should be conducted to examine whether the same ratings and classifications of words apply to other age groups. Dominance ratings should also be established in future. Finally, a future study could ask the same participants to rate the words according to different dimensions and compare the results to those of the present study.

## Supporting Information

S1 Table Ratings of 160 Chinese words. (XLSX)
